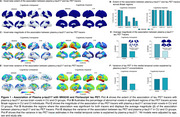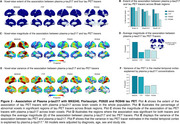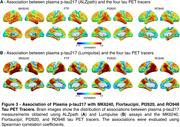# Head‐to‐Head evaluation of plasma *p*‐tau217 associations with MK6240, Flortaucipir, PI2620, and RO948 tau PET tracers

**DOI:** 10.1002/alz70862_110043

**Published:** 2025-12-23

**Authors:** Pamela C.L. Ferreira, Tevy Chan, Bruna Bellaver, Guilherme Povala, Guilherme Bauer‐Negrini, Emma Ruppert, Carolina Soares, Cynthia Felix, Andreia Rocha, Marina Scop Madeiros, Livia Amaral, Juli Cehula, Firoza Z Lussier, Joseph C. Masdeu, Dana L Tudorascu, Thomas K Karikari, David N. soleimani‐Meigooni, Juan Fortea, Val J Lowe, Hwamee Oh, Belen Pascual, Brian A. Gordon, Pedro Rosa‐Neto, Suzanne L. Baker, Tharick A Pascoal

**Affiliations:** ^1^ University of Pittsburgh, Pittsburgh, PA USA; ^2^ McGill University, Montreal, QC Canada; ^3^ Houston Methodist Research Institute, Houston, TX USA; ^4^ Lawrence Berkeley National Laboratory, Berkeley, CA USA; ^5^ Sant Pau Memory Unit, Hospital de la Santa Creu i Sant Pau, Institut de Recerca Sant Pau ‐ Universitat Autònoma de Barcelona, Barcelona Spain; ^6^ Mayo Clinic, Rochester, MN USA; ^7^ Brown University, Providence, RI USA; ^8^ Washington University in St. Louis, School of Medicine, St. Louis, MO USA; ^9^ University of Pittsburgh School of Medicine, Pittsburgh, PA USA

## Abstract

**Background:**

Tau phosphorylation and aggregation are hallmark features of Alzheimer's disease pathology(AD). Previous studies have shown that plasma phosphorylated tau(*p*‐tau) at threonine 217 can detect AD‐related tau tangle pathology. However, the head‐to‐head association between *p*‐tau217 and different tau PET tracers remains unexplored. Here, we conducted a head‐to‐head comparison of the association between plasma *p*‐tau217 and four distinct tau PET tracers[MK6240, Flortaucipir(FTP), PI2620, RO948].

**Method:**

We studied 338 individuals across the AD continuum from the HEAD study[190 cognitively unimpaired elderly(CU), 109 with mild cognitive impairment(MCI), and 39 with dementia] with available FTP, MK6240, and plasma *p*‐tau217 measured by ALZpath assay. A subset of 64 individuals(28 CU, 25 MCI, and 11 dementia) also had PI2620 and RO948, and *p*‐tau217 measured by Lumipulse. The strength of the association was determined using t‐value maps from regression models testing the association between each tau PET tracer and plasma *p*‐tau217, and the extent was measured by the percentage of significant voxels(t‐value>3.2). R‐square metric was used to determine how much of the variance in tau PET tracer estimates was explained by plasma *p*‐tau217 concentrations. Associations between *p*‐tau217 with tau PET were evaluated using the Spearman test.

**Result:**

We found that in CU, both the extent and magnitude of the association with plasma *p*‐tau217 were higher for MK6240 compared to FTP (Figures 1A‐D). Plasma *p*‐tau217 explained a greater proportion of the variance in MK6240 than in FTP (Figures 1E‐F). In CI, the extent and magnitude of the association were similar for MK6240 and FTP. Additionally, plasma *p*‐tau217 explained a similar proportion of the variance in MK6240 and FTP. Using the subset of individuals who had all tau tracers, MK6240 showed a slightly higher extent of association with *p*‐tau217, followed by FTP, PI2620, and RO948 (Figures 2A‐B). The magnitude of association was stronger for MK6240, followed by PI2620, RO948, and FTP (Figures 2C‐D). Plasma *p*‐tau217 explained a greater proportion of the variance in MK6240, followed by FTP, RO948, and PI2620 (Figures 2E‐F). We compared the associations between different *p*‐tau217 assays, and both assays presented similar associations with all tau PET tracers (Figure 3).

**Conclusion:**

In summary, our results indicate that the four tau PET tracers exhibit robust associations with plasma *p*‐tau217 in similar topographical regions.